# Papillary fibroelastoma of the aortic valve in association with rheumatic heart disease: a case report

**DOI:** 10.1186/s13019-016-0410-6

**Published:** 2016-01-16

**Authors:** Jun Shi, Zhi-xuan Bai, Ben-gui Zhang, Ying-qiang Guo

**Affiliations:** Department of Cardiovascular Surgery, West China Hospital, Sichuan University, 37 Guoxue Xiang St, Chengdu, Sichuan China

**Keywords:** Cardiac tumors, Papillary fibroelastoma, Rheumatic heart disease

## Abstract

**Background:**

Papillary fibroelastoma (PFE) is a rare primary cardiac neoplasm that is usually discovered incidentally at autopsy or during cardiac surgery. PFE combined with rheumatic heart disease (RHD) is extremely rare, and only a few cases have been reported. Additionally, the growth rate of the tumor is unknown.

**Case Presentation:**

Here, we present a very rare case of PFE of the aortic valve combined with RHD, which were identified in a female patient who survived for 5 years without surgical intervention, and who subsequently underwent successful surgical treatment.

**Conclusions:**

PFEs may be generally slow-growing tumors, however, the better treatment of choice may be surgery because it produces good curative effects with very low risk of complications, while preventing serious disease consequences.

## Background

Although papillary fibroelastoma (PFE) is rare, it is the second most common primary cardiac neoplasm, accounting for 4.4 % to 8 % of all tumors of the heart [1]. PFE is usually discovered incidentally at autopsy or during cardiac surgery. With the advent of higher-resolution imaging technologies, especially transesophageal echocardiography, cases of PFE are being recognized more frequently [2]. However, the etiology of PFE and the time that it takes to develop are both unknown.

Here, we present the case of a 55-year-old female patient who had a PFE of the aortic valve in combination with rheumatic heart disease (RHD). She was diagnosed with RHD in 2010. Although transthoracic echocardiography revealed a mass on the non-coronary cusp at that time, the patient initially refused surgery for economic reasons. A surgical intervention was ultimately performed 5 years later. In the intervening time, she did not develop any symptoms related to the PFE, which did not grow significantly. We view this case as being instructive, both because PFE rarely appears in combination with RHD, and because few clinicians are able to observe the natural history of surgically untreated PFE over such a long interval of time.

### Case Presentation

**Fig. 1:**
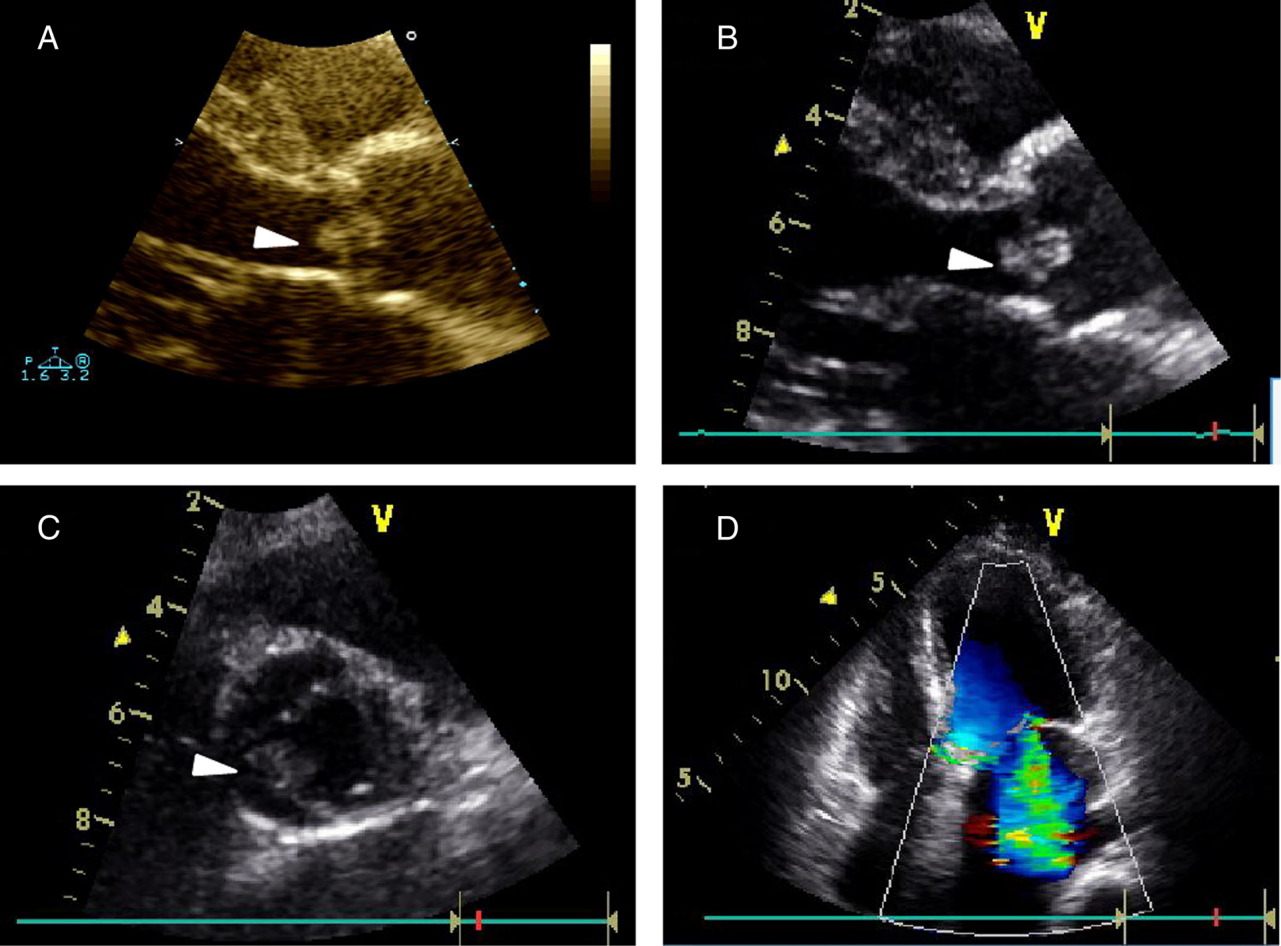
Transthoracic echocardiography. **a**, parasternal long-axis view shows a round mass (6 × 5 mm, arrow) on the aortic side of the non-coronary cusp of the aortic valve in 2010. **b**, parasternal long-axis view shows a round mass (6 × 5 mm, arrow) on the aortic side of the non-coronary cusp of the aortic valve in 2015. **c**, parasternal short-axis view shows a round mass (arrow) on the aortic side of the non-coronary cusp of the aortic valve in 2015. **d**, Apical 4 chamber view shows moderate mitral regurgitation

A 50-year-old woman was first admitted to our hospital in 2010, when transthoracic echocardiography (TTE) revealed a mass on the non-coronary cusp for the first time (Fig. 1), and electrocardiogram showed sinus rhythm. The patient had valvular heart disease that required surgery, which we strongly recommended as the measure to treat the disease as well as to confirm the diagnosis of the mass. However, the patient refused, mainly because of economic reasons. During the five subsequent years, we were unable to contact the patient until she returned in 2015.

In 2015, the 55-year-old woman who had a New York Heart Association (NYHA) functional class of III was admitted with the diagnosis of RHD presented as exertional shortness of breath (duration, 6 years) accompanied by occasional palpitations and dizziness. The patient denied any chest pain, orthopnea, or paroxysmal nocturnal dyspnea. She denied any history of cardiac tumors, coronary artery disease, pulmonary disease, or cancer, as well as smoking, alcohol consumption, or use of illicit drugs. Physical examination showed blood pressure of 130/65 mmHg, temperature of 36.7 °C, and heart rate of 76 beats/min. Findings of chest radiography were normal, while electrocardiography detected atrial fibrillation. The results of routine blood laboratory investigations were unremarkable. TTE revealed moderate mitral stenosis (mitral valve area, 1.1 cm^2^), moderate mitral regurgitation, mild tricuspid regurgitation, mild to moderate aortic valve regurgitation, and a round mass (6 × 5 mm) on the aortic side of the non-coronary cusp (Fig. 1).

She admitted occasionally taking diuretics on her own accord without medical recommendation, but denied taking any anticoagulants during the past five years.

**Fig. 2:**
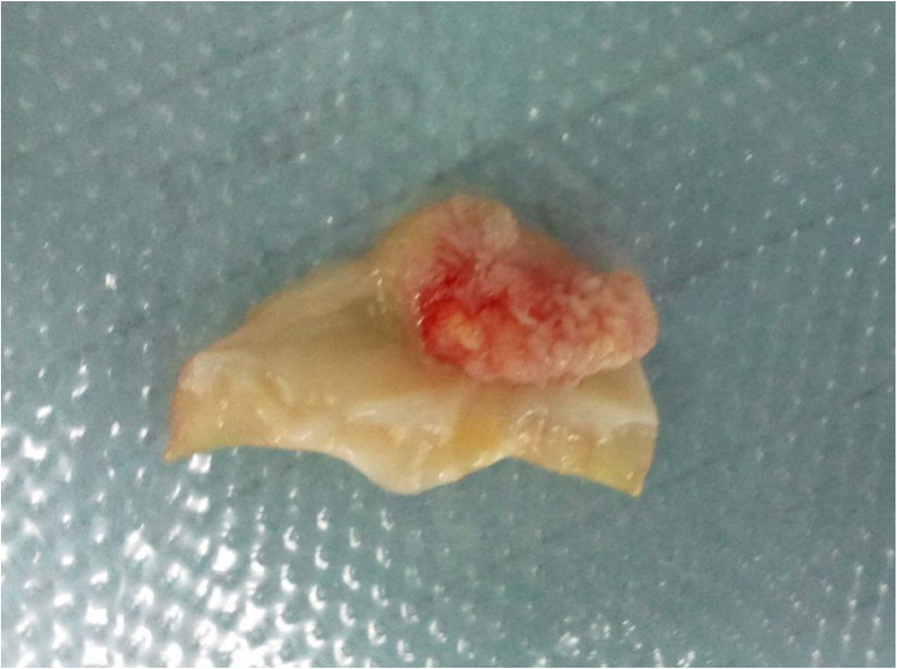
Gross specimen of the resected mass and the non-coronary cusp of the aortic valve

The diagnosis of RHD was clear, and the aortic valve mass was considered to be an inflammatory mass, a myxoma, or a fibroelastoma. Through median sternotomy, a cardiopulmonary bypass was established in the conventional manner, and the mitral valve was found to have grossly thickened and calcified leaflets with commissural fusion. The aortic valve was also thickened, and a flower-like mass with multiple papillary fronds (8 × 8 mm) was found on the aortic side of the non-coronary cusp (Fig. 2). Because of the rheumatic change and aortic regurgitation, the valve was judged to be unsuitable for repair and was excised along with the tumor. The mitral valve and aortic valve were both replaced with St. Jude bileaflet mechanical valves. The tricuspid valve was repaired; a bipolar radiofrequency ablation procedure was performed to treat the atrial fibrillation. The case’s postoperative course progressed smoothly, and TTE demonstrated proper functioning of the prosthesis.

**Fig. 3:**
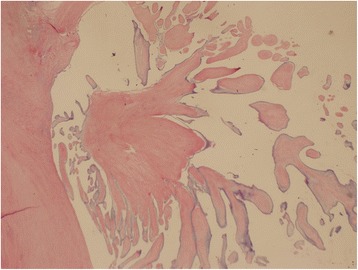
Hematoxylin and eosin stain (original magnification × 20)

**Fig. 4:**
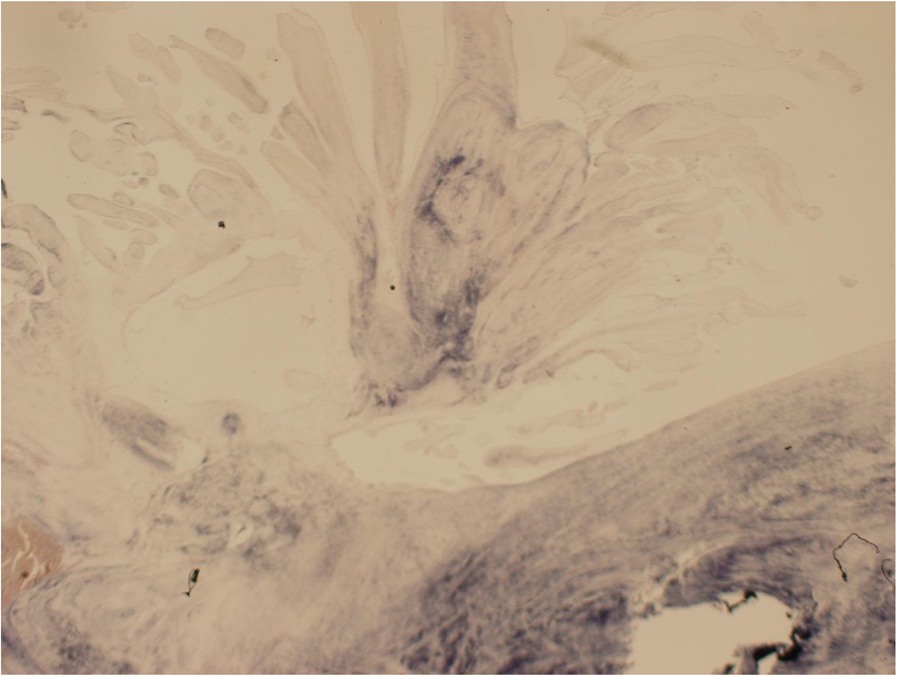
Verhoeff's elastic stain (original magnification × 20)

Histopathological examination of the resected tumor revealed avascular papillomas with a single layer of endocardial cells covering the papillary surface. The connective tissue of PFE contains mature collagen with irregular elastic fibers that are oriented longitudinally (Figs. 3 and 4). The appearance of the mitral valve and aortic valve was consistent with healed rheumatic valve disease.

## Conclusions

PFE is a rare, benign cardiac neoplasm with an estimated frequency of 0.021 % in autopsy series [3]. PFE is the second most common type of benign tumor of the heart, following myxoma [4]. PFE tumors are predominantly located at valvular surfaces. The aortic valve is the most commonly involved valve (44 %), followed by the mitral valve (35 %), tricuspid valve (15 %), and pulmonary valve (8 %). The other reported sites of PFE involvement are the left atrium, atrial septum, right atrium, and right ventricle [5].

The natural history of PFE has not been defined because longitudinal follow-up studies have not been performed. In this unusual case, the patient provides us with the opportunity to learn about the natural history of PFE indirectly. The tumor did not grow significantly during the 5-year period, indicated that PFEs may be generally slow-growing tumors.

Although the cause of PFE remains unknown, Kurup and colleagues have noted that PFE could potentially be related to cardiovascular intimal trauma. A history of heart surgery or radiation to the chest may cause PFE [6]. Regarding the patient in this report, the only potential endocardial irritation was RHD. However, further investigation is still needed to determine whether RHD could cause PFE.

Most PEFs have been asymptomatic, and they have usually been discovered incidentally at the time of echocardiography, cardiac surgery, or autopsy [7]. Patients with symptomatic PFE can experience a variety of symptoms. The clinical manifestations depend on many factors, including the tumor's mobility, location, size, and tendency for embolization. The main cause of symptoms is thrombosis, such as cerebrovascular accident, or obstruction of the coronary artery, which results in cardiovascular symptoms and events such as chest pain, myocardial infarction, and even sudden death (in severe cases) [8].

 For symptomatic patients, especially for those with mobile tumor, surgical resection with or without valve replacement is recommended. These patients are at high risk for thrombosis, which may be life-threatening, whereas surgical removal poses low risk and the outcome is excellent [9, 10]. However, surgical resection remains controversial for patients without symptoms. Ngaage et al. supported prompt surgical resection in asymptomatic PFE cases because of potential life-threatening complications [7]. On the other hand, Klarichhas et al. have reported that PFEs not treated with surgery did not result in increased mortality, but may be associated with higher risk of neurologic events. Hence, for patients who are not surgical candidates or refuse surgery, anticoagulation should be considered to prevent clot formation on PFE surface, while the patients should be closely followed-up [11].

During the 5-year period passed between her initial presentation and the second admission, our patient did not take anticoagulants and did not suffer any thrombosis, which, however, does not mean that it is completely safe to leave PFE without treatment. In our opinion, the better treatment of choice may be surgery because it produces good curative effects with very low risk of complications, while preventing serious disease consequences. Still, further studies are required to determine the best treatment for PFEs.

### Consent

Written informed consent was obtained from the patient for the publication of this case report and any accompanying image. A copy of the written consent is available for review by the Editor-in-Chief of this journal.

## Abbreviations

PFE: Papillary fibroelastoma
RHD: Rheumatic heart disease
TTE: Transthoracic echocardiography
